# High temperature rise dominated cracking mechanisms in ultra-ductile and tough titanium alloy

**DOI:** 10.1038/s41467-020-15772-1

**Published:** 2020-04-30

**Authors:** L. Choisez, L. Ding, M. Marteleur, H. Idrissi, T. Pardoen, P. J. Jacques

**Affiliations:** 1 Institute of Mechanics, Materials and Civil Engineering, IMAP, UCLouvain, Place Sainte Barbe, 2, B-1348 Louvain-la-Neuve, Belgium; 2 Department of Physics, Electron Microscopy for Materials Science (EMAT), University of Antwerp, Groenenborgerlaan 171, 2020 Antwerpen, Belgium

**Keywords:** Mechanical properties, Metals and alloys

## Abstract

Extensive use of titanium alloys is partly hindered by a lack of ductility, strain hardening, and fracture toughness. Recently, several *β*-metastable titanium alloys were designed to simultaneously activate both transformation-induced plasticity and twinning-induced plasticity effects, resulting in significant improvements to their strain hardening capacity and resistance to plastic localization. Here, we report an ultra-large fracture resistance in a Ti-12Mo alloy (wt.%), that results from a high resistance to damage nucleation, with an unexpected fracture phenomenology under quasi-static loading. Necking develops at a large uniform true strain of 0.3 while fracture initiates at a true fracture strain of 1.0 by intense through-thickness shear within a thin localized shear band. Transmission electron microscopy reveals that dynamic recrystallization occurs in this band, while local partial melting is observed on the fracture surface. Shear band temperatures of 1250–2450 °C are estimated by the fusible coating method. The reported high ductility combined to the unconventional fracture process opens alternative avenues toward Ti alloys toughening.

## Abstract (web summary)

Specific titanium alloys combine transformation-induced plasticity and twinning-induced plasticity for improved work hardening. Here, the authors show that these alloys also have an ultra-large fracture resistance and an unexpected fracture mechanism via dynamic recrystallization and local melting in a deformation band.

## Introduction

Recently, a class of $\beta$-metastable Ti alloys exhibiting exceptional levels of strain hardening and uniform elongation resulting from the synergetic activation of TRIP (TRansformation-Induced Plasticity) and TWIP (TWinning-Induced Plasticity) effects has attracted major attention^1–8^. However, the post-necking and fracture properties of such alloys together with the underlying failure mechanisms have never been addressed in the literature, even though toughness and fracture resistance are critical properties for numerous structural applications.

The mechanical performances of structural materials can be defined by several properties. The yield stress, $\sigma _{\mathrm{y}}$, sets the resistance to the onset of plastic deformation. The uniform elongation, ${\it{\epsilon }}_{\mathrm{u}}$, (expressed hereafter as true strain) quantifies the resistance to plastic localization and is directly related to the strain hardening capacity, with $\sigma _{\mathrm{u}}$ being the corresponding stress and $W_{\mathrm{n}} = \mathop {\smallint }\nolimits_0^{{\it{\epsilon }}_{\mathrm{u}}} \sigma d{\it{\epsilon }}$ the work per unit volume dissipated until necking, sometimes called “toughness”. The true fracture strain, ${\it{\epsilon }}_{\mathrm{f}}$, characterizes the resistance to damage, with $\sigma _{\mathrm{f}}$ being the true fracture stress and $W_{\mathrm{f}} = \mathop {\smallint }\nolimits_0^{{\it{\epsilon }}_{\mathrm{f}}} \sigma d{\it{\epsilon }}$ the work per unit volume dissipated until final fracture in the fracture zone. The stress intensity factor, $K_{{\mathrm{IC}}}$, the critical *J* integral, $J_{{\mathrm{IC}}}$, and/or corresponding critical crack tip opening displacement, $\delta _{\mathrm{c}}$, characterize the resistance to crack initiation in a pre-cracked material, known as fracture toughness.

The development of alloys over the last decades has been often driven by the quest of increased strength levels $\sigma _{\mathrm{y}}$ and/or $\sigma _{\mathrm{u}}$ (by playing on hardening mechanisms) and, more recently, of enhanced strength-ductility balance $\sigma _{\mathrm{u}} - {\it{\epsilon }}_{\mathrm{u}}$(by boosting the strain hardening capacity). However, microstructure engineering studies to tackle damage and fracture resistance are more rarely reported.

Depending on the stress state and on the mechanical properties and microstructure, plastic localization or damage accumulation is predominant and dictates the onset of fracture of the material^9^. Plastic localization usually dominates in materials with low strain hardening like pure metals, as cp-Ti^10^. In uniaxial tension, macroscopic plastic localization occurs first through the necking process when the Considère criterion is reached, i.e., when the cross section reduction is not compensated anymore by the strain hardening. The initially diffuse localization can then continue through a more localized shear banding process until the material fails by pure plastic flow without any occurrence of damage as in pure metals with no inclusions, or until damage initiates in the localized band as in pure metals containing non-metallic inclusions^11^. In materials with almost inexistent work hardening like nanocrystalline materials or bulk metallic glasses, shear banding is often activated very soon after the onset of plasticity, resulting in a catastrophic failure^12,13^. On the other hand, damage accumulation usually dominates failure (or occurs soon after the onset of necking) in the most recent advanced metallic alloys (e.g., high strength Ti alloys like $\beta$-CEZ, TRIP and TWIP steels, Q&P steels,…), where numerous interfaces are integrated within the microstructure to increase strength, but often at the expense of ductility^14–18^. The introduction of a large density of interfaces is in principle detrimental as it provides more opportunities for damage nucleation before plastic localization, resulting from the strain incompatibility at these interfaces. The accumulated porosity can partly counteract the strain-hardening capacity and accelerate plastic localization, or even directly lead to failure if void coalescence occurs before any plastic instability^9^. However, Wang et al.^19^ recently postulated that the activation of the TWIP effect (mechanically induced nanotwins) could improve the fracture resistance of TRIP–TWIP steels by acting as a barrier against the growth of nanovoids nucleated at the interface of martensite transformed from austenite (TRIP effect). Therefore, a simultaneous activation of TRIP and TWIP effects in Ti alloys could possibly have the same positive influence on the resistance to damage. This is certainly essential with respect to numerous applications requiring high fracture toughness, not only for critical structural components but also in some forming processes limited by damage and cracking issues.

Here, we report the large damage resistance, as quantified by the work of fracture $W_{\mathrm{f}}$ and confirmed by an estimation of the fracture toughness, of the generic Ti-12Mo (wt.%) alloy despite the huge density of interfaces originating from the TRIP and TWIP effects. In other words, this alloy not only involves a high resistance to plastic localization in terms of high ${\it{\epsilon }}_{\mathrm{u}}$ and high ${\it{\epsilon }}_{\mathrm{u}}\sigma _{\mathrm{u}}$ indicators as proved in earlier recent reports^1,2^, but also an exceptional high resistance to fracture that overtops classical titanium alloys. This is a unique combination of performances allowing both high formability, high crash resistance and high resistance to cracking. In addition, a fundamental study of the underlying failure mechanisms demonstrates large temperature rise during the quasi-static deformation of the investigated alloy with multiple local modifications of the microstructure. The resulting damage and cracking mechanisms are also found to be totally unusual for a polycrystalline alloy exhibiting a high work-hardening rate.

## Results

### Tensile properties

**Fig. 1:**
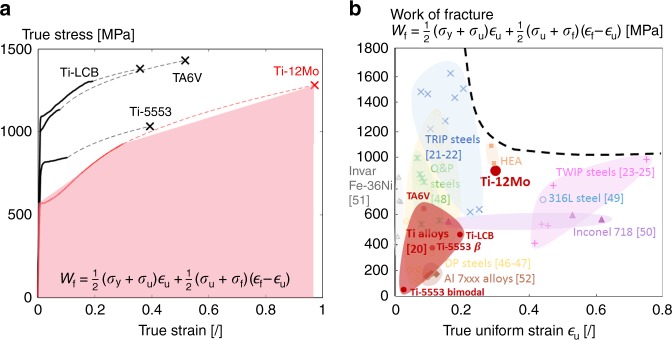
Post-necking properties of the Ti-12Mo (wt.%) alloy compared with other high strength alloys^20–25,46–52^. **a** True stress-true strain curves of Ti-12Mo compared with classical Ti alloys. A cross indicates the true fracture stress and true fracture strain. A dashed line represents the extrapolation between the true tensile curve recorded up to necking and the true fracture stress—true fracture strain. The red colored area represents the integration performed to estimate the work of fracture $W_{\mathrm{f}}$, which is used as an indicator of the fracture toughness. **b** Comparison of the work of fracture $W_{\mathrm{f}}$ versus true uniform strain ${\it{\epsilon }}_{\mathrm{u}}$ of several alloys. The major families of alloys are grouped by colored circles. A black dashed line indicates the limit of the best combination of both properties. Ti-12Mo alloy is located among the best materials on the map, while its outstanding mechanical properties with respect to classical Ti alloys are clearly demonstrated. The mechanical properties of the HEA and some DP steels result from internal unpublished PhD theses.

Figure 1a presents the true stress-true strain tensile curves of the Ti-12Mo (wt.%) alloy (see in “Methods” for material information) compared with other classical Ti alloys, i.e., TA6V, Ti5553, and Ti LCB alloys^20^. While the yield strength of the Ti-12Mo alloy is lower, the true uniform strain is by far the largest since the macroscopic strain localization has been postponed owing to a high strain-hardening capacity^1,2^. Figure 1a also illustrates the magnitude of the properties at fracture. The true fracture stress ($\sigma _{\mathrm{f}} = F_{\mathrm{f}}/A_{\mathrm{f}}$) and true fracture strain (${\it{\epsilon }}_{\mathrm{f}} = \ln A_0/A_{\mathrm{f}}$) were computed from the last force value $F_{\mathrm{f}}$ recorded before fracture, from the fracture area $A_{\mathrm{f}}$ and from the initial section area of the tensile specimen $A_0$. The fracture area was estimated from the smallest width at fracture, measured from the reconstituted fractured parts, and from the thickness of the neck, averaged over ten measurements along the width of the polished fractured parts. The tensile properties between the neck and the fracture point are extrapolated as a dashed line according to a power law relationship. The true fracture strain of the Ti-12Mo alloy is twice larger than the one of other reference Ti alloys with a comparable fracture stress. The association of such large levels of strength and ductility leads to exceptionally high levels of work of fracture $W_{\mathrm{f}}$, representing the total amount of energy to be brought in the fracture zone to activate ductile failure, approximated here by $\frac{1}{2}( {\sigma _{\mathrm{y}} + \sigma _{\mathrm{u}}} ){\it{\epsilon }}_{\mathrm{u}} + \frac{1}{2}(\sigma _{\mathrm{u}} + \sigma _{\mathrm{f}})({\it{\epsilon }}_{\mathrm{f}} - {\it{\epsilon }}_{\mathrm{u}})$ in order to allow the comparison with results from the literature. The work of fracture of Ti-12Mo, 865 MJ.m^−3^, significantly overtops the one of other classical Ti alloys, i.e., 360 MJ.m^−3^ for Ti-5553, 440 MJ.m^−3^ for Ti-LCB, and 620 MJ.m^−3^ for TA6V. The properties of the present Ti-12Mo alloy are also compared with other high performance metallic materials in Fig. 1b in terms of combination of $W_{\mathrm{f}}$ and ${\it{\epsilon }}_{\mathrm{u}}$. This graph combines the two indicators that dictate the resistance to failure of ductile metals: $W_{\mathrm{f}}$ for damage controlled fracture and ${\it{\epsilon }}_{\mathrm{u}}$ for plastic localization controlled fracture. The figure shows that, more than largely overpassing classical titanium alloys, the present Ti-12Mo alloy exhibits a combination of $W_{\mathrm{f}}$ and ${\it{\epsilon }}_{\mathrm{u}}$ comparable to the currently best materials like specific TRIP steels^21,22^, high entropy alloys and TWIP steels^23–25^. The high ductility and failure resistance of Ti-12Mo, combined with the well-known interesting properties of Ti alloys (low density, high resistance to corrosion, biocompatibility), increases the range of potential applications of Ti alloys.

Moreover, a high level of work of fracture is a good indicator of anticipated large fracture toughness. The fracture toughness $J_{{\mathrm{IC}}}$ is indeed defined as the work per unit area spent in the fracture process zone (FPZ). The work of separation in the FPZ is given by $\mathop {\smallint }\nolimits_0^{u_{\mathrm{f}}} \sigma du$ or $X_0\mathop {\smallint }\nolimits_0^{{\it{\epsilon }}_{\mathrm{f}}} \sigma d{\it{\epsilon }}$, where $u$ is the displacement at the boundaries of the FPZ, $\sigma$ the opening stress and $X_0$ the thickness of the FPZ^26^. The fracture toughness is thus directly related to the work of fracture, *J*_IC_ = *X*_0_*W*_f_. However, a limitation to this reasoning is the change of stress triaxiality between a uniaxial tensile test and the propagation of a crack. The work of fracture measured in uniaxial tension will be higher than the work of fracture evaluated at the tip of the crack, and the stress concentration at the tip of a crack could also trigger a change of fracture mechanism. Consequently, a better estimation of the fracture toughness is given below by the critical crack tip opening displacement $\delta _{\mathrm{c}}$.

### Fracture toughness

The critical crack tip opening displacement, $\delta _{\mathrm{c}}$, was estimated from the propagation of pre-cracks in three double edge notched tensile specimens (DENT) under near plane stress conditions as $\delta _c = 146 \pm 29$ µm. Based on a Shih factor $d_{\mathrm{n}}$ in plane stress equal to 0.4^27^, the fracture toughness of Ti-12Mo can be estimated as $J_{{\mathrm{IC}}} = \frac{{\sigma _{\mathrm{y}}\delta _{\mathrm{c}}}}{{d_{\mathrm{n}}}}\sim 220\,{\mathrm{kJ}.\mathrm{m}^{-2}}$, or $K_{\mathrm{IC}} = \sqrt {\mathit{EJ}_{\mathit{IC}}} \sim 140\,{\mathrm{MPa.m}}^{\frac{1}{2}}$. Even though the fracture toughness derived from $\delta _{\mathrm{c}}$ is only a rough estimation, the obtained value for Ti-12Mo is larger than the fracture toughness of classical Ti alloys such as TA6V($K_{{\mathrm{IC}}}\sim 40{-}100\,{\mathrm{MPa.m}}^{\frac{1}{2}}$)^28,29^.

### Damage resistance

**Fig. 2:**
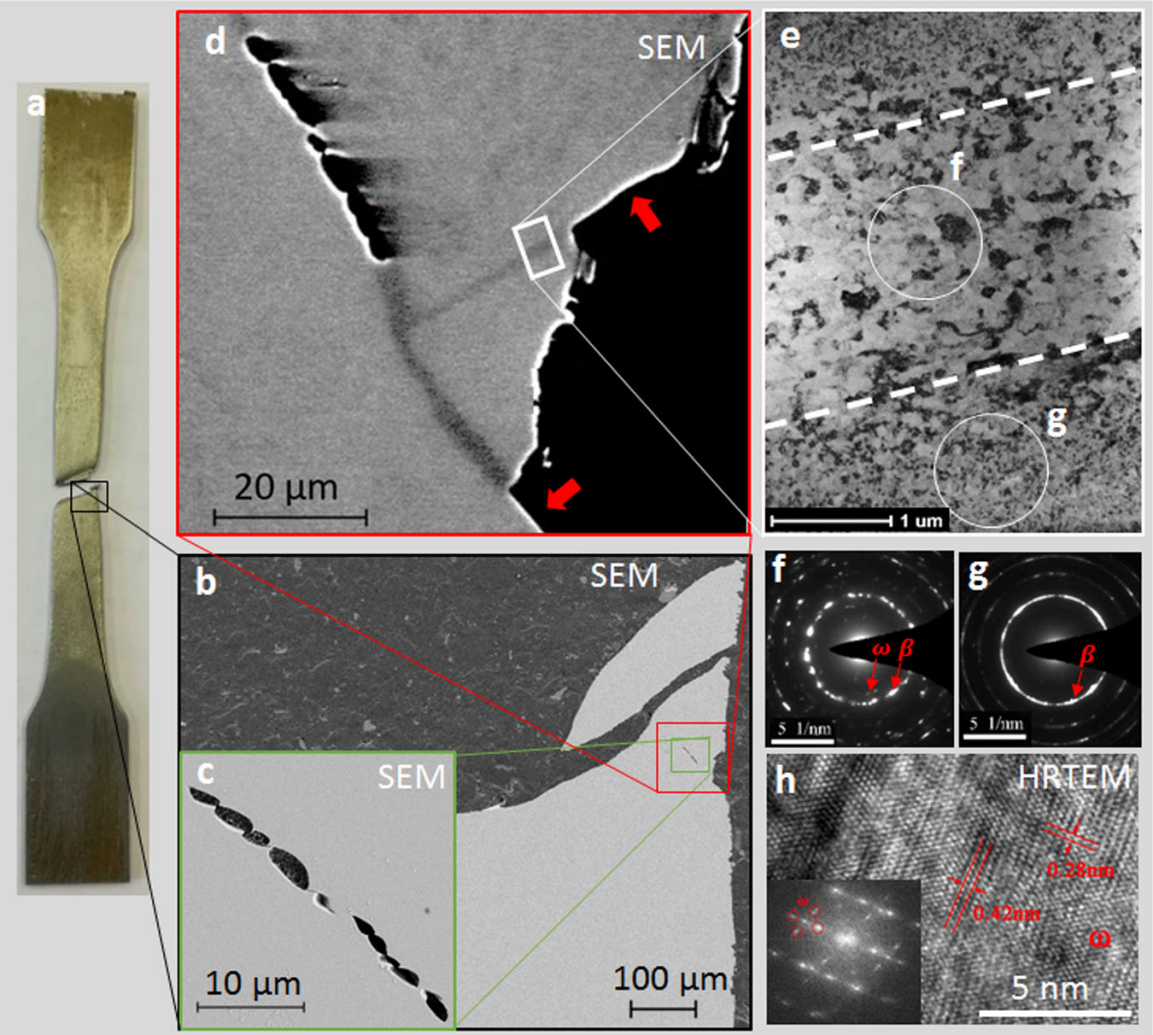
Damage in localized shear bands. **a** After testing, a fractured tensile specimen was polished to a third of its thickness. **b** Secondary electrons SEM micrograph shows that there is almost no cavity in the fractured tensile specimen. **c** The only cavities observed are aligned in a micro-crack. **d** Backscattered electrons contrast shows that the cavities are formed within localized shear bands located underneath the fracture surface. The two red arrows indicate the shear offsets resulting from the shearing process. The white rectangle indicates the location where the TEM foil was extracted by FIB. **e** TEM bright-field micrograph revealing the microstructure inside the band (equiaxed grains) and outside the band (nanoblocks). The limits of the band are highlighted by dotted lines. **f–g** Selected area electron diffraction (SAED) patterns taken inside the band and outside the band, respectively. While $\omega$ spots are detected in **f** together with the $\beta$ spots, the $\omega$ spots are not visible in **g**. **h** High-resolution TEM micrograph revealing the presence of $\omega$ phase inside the band (**f**). The HRTEM micrograph was taken along the <111> zone axis of the $\beta$ phase. The measured distances of 0.42 and 0.28 nm within the precipitate correspond to the interplanar spacings of the (110) and (100) planes of the $\omega$ phase.

The origin of the exceptional ductility of the Ti-12Mo alloy is associated with its surprisingly large resistance to damage nucleation. While no cavities could be observed through the thickness of fractured specimens by X-ray computed tomography (see Supplementary Fig. 1), SEM micrographs of the fractured specimens revealed the presence of some cavities, 1–3 µm thick and 2–6 µm long, aligned as a micro-crack after the void coalescence step underneath the fracture surface (see Fig. 2a–d). The few cavities found inside the fractured specimen stand in line within localized deformation bands of same thickness (1–3 µm). The two shear offsets indicated by the red arrows in Fig. 2d show that these bands result from an intense shearing process. The nucleation of these cavities occurs just before fracture, as no cavity was detected in a tensile specimen deformed to a true strain of 1.03, and stopped just before fracture (see Supplementary Fig. 2).

### Transmission electron microscopy (TEM) analysis

A TEM specimen was machined perpendicularly to the band by focused ion beam (FIB) milling (as illustrated by the white rectangle in Fig. 2d) in order to investigate the microstructure changes occurring within these bands. The bright-field TEM micrograph of Fig. 2e as well as the selected area electron diffraction (SAED) patterns of Fig. 2f, g illustrate that the band presents a different microstructure from the surrounding. Out of this band, very small nanoblocks of about 30 nm can be observed, in agreement with the continuous diffraction rings shown in the corresponding SAED. Within the band, both TEM contrast and SAED show the presence of well-defined small equiaxed grains (118 ± 24 nm). Furthermore, the SAED patterns of Fig. 2f, g do not present exactly the same diffraction spots. Indeed, while no other spots than the ones corresponding to the $\beta$ phase can be found in Fig. 2g, extra spots corresponding to the athermal *ω* ($\omega _{{\mathrm{ath}}}$) are present in Fig. 2f. The presence of nanoprecipitates of $\omega _{{\mathrm{ath}}}$ has also been confirmed by high-resolution TEM as shown in Fig. 2h. This means that nanoprecipitates of $\omega _{{\mathrm{ath}}}$ initially present in the $\beta$ phase of quenched Ti-12Mo (wt.%)^1^ were dissolved by the large level of plastic deformation, but appeared again in the band leading to fracture.

**Fig. 3:**
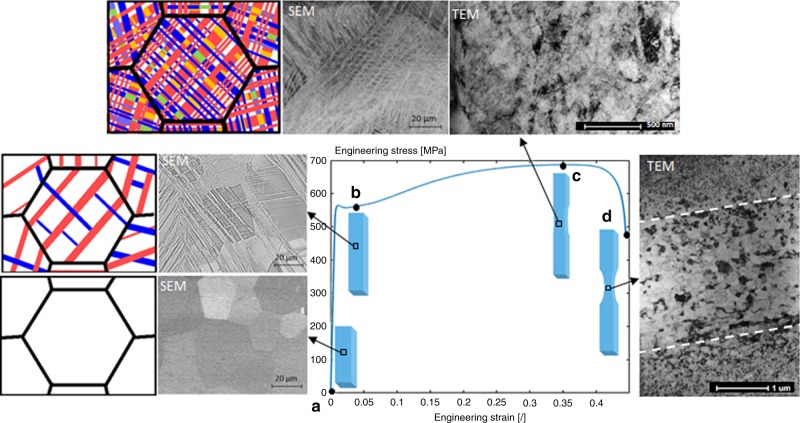
Evolution of the microstructure during deformation. **a** The initial microstructure is constituted of equiaxed grains 40 ± 9 µm in size. **b** At a true strain of 0.05, several deformation “bands” are formed within the grains during deformation resulting from the simultaneous activation of the TRIP and TWIP effects. **c** At necking, the density of deformation “bands” is very large, with multiple secondary deformation “bands” formed in the primary ones. A TEM micrograph shows that the microstructure is constituted by nanoblocks about 50–100 nm size containing a huge density of dislocations. **d** At a deformation level close to the fracture strain, the microstructure is further refined together with larger equiaxed grains in the localized shear bands, indicated in the figure by white dashed lines.

The evolution of the microstructure during the tensile deformation is thus schematically represented in Fig. 3. While the initial grains of Ti-12Mo are quite large (40 ± 9 µm) (see Fig. 3a), the high density of mechanically induced twins and martensite dynamically reduces the size of apparent grains^2^. Figure 3b presents the microstructure at a true strain of 0.05, where the size of the $\beta$ untwinned blocks is already reduced to about 4 µm. TEM analyses carried out for small levels of deformation showed the formation of submicron “blocks” inside the twins as a result of the activation of secondary martensite transformation and twinning mechanisms^1,2,6^. At necking, nanoblocks about 50–100 nm in size can be observed as illustrated in Fig. 3c. The size of these nanoblocks is further decreased for a larger level of deformation near the fracture surface (where the deformation level is close to the true fracture strain (see Fig. 3d). A few localized shear bands eventually form and consist of larger equiaxed grains containing again $\omega _{{\mathrm{ath}}}$ nanoprecipitates, as already presented in Fig. 2.

### Fractography

**Fig. 4:**
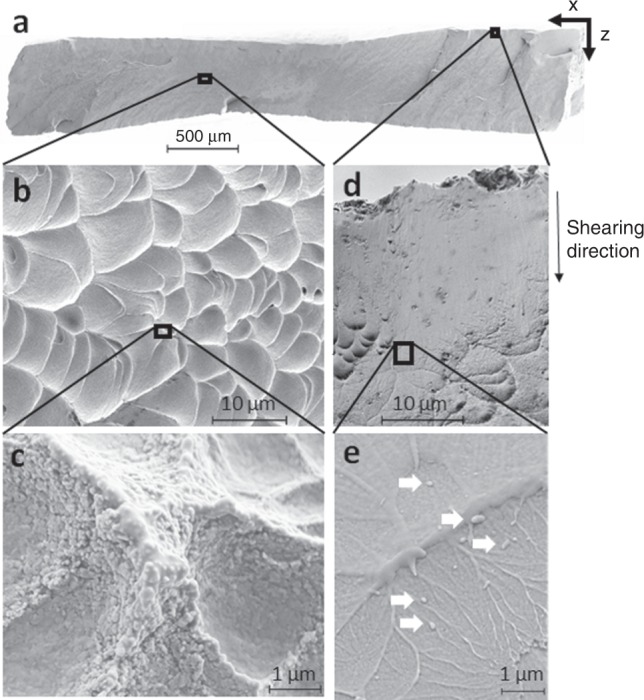
SEM micrographs of the fracture surface at multiple length-scales. **a** Projection of the entire fracture surface in the section of the tensile specimen. **b** Representative flat shaped dimples covering most of the fracture surface. **c** Magnified view of a dimple wall. The cap of the wall appears as molten and resolidified. **d** Overview of the stretched surface sheared along the *z* axis, covering one side of the fracture surface along the *x* axis. **e** Magnified view of the transition between the stretched surface and the dimples. The observed pattern looks like a Taylor meniscus pattern, which only forms in viscous materials. The white arrows indicate the presence of resolidified droplets.

Figure 4 provides a multiscale characterization of the fracture surface, with an overall view in Fig. 4a. It mostly consists of flat dimples such as shown in Fig. 4b, with a diameter of 3.1 ± 0.1 µm and an average height of the walls of the order of half of the thickness of the localized shear bands (1.5 ± 0.3 µm). The observation of the walls of the dimples at high magnification in Fig. 4c reveals a granular appearance except at the top of the wall which exhibits a smooth, viscous-like appearance, resulting from melting and resolidification. On the other hand, Fig. 4d shows that a stretched zone is present at the edge of the fracture surface, resulting from the shearing of the specimen at 45° from the tensile axis and from the thickness direction (*z* axis). This zone is present along the entire width of the specimen (*x* direction). The length of this stretched surface, or shear offset, was estimated to be 27 ± 8 µm. Figure 4e shows that a specific fracture pattern can be observed at the transition between the stretched surface and the area covered with the dimples. This pattern is very similar to a so-called Taylor meniscus instability pattern^30,31^, which consists of several interconnected ridges caused by a finger-like perturbation growth starting at the edges of the specimen.

**Fig. 5:**
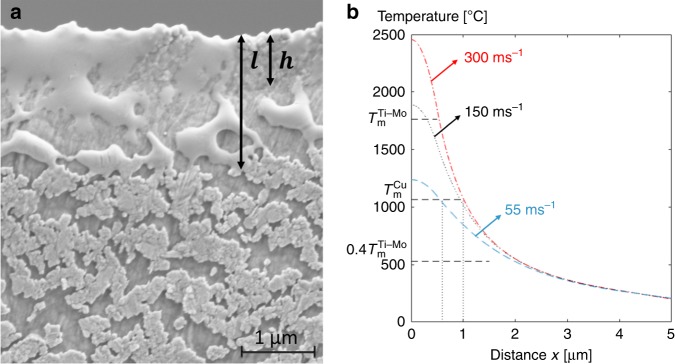
Temperature rise in the localized band. **a** SEM micrograph of the Cu coating on the tensile specimen after fracture. The coating melted over a distance *l* on the fractured specimen. A larger wettability is however observed over a distance *h*, where the specimen is deformed the most, which demonstrates a different thermomechanical path of this zone from the rest of the sample. **b** Profiles of the highest temperature reached at a normal distance *x* from the center of the shear band, for shearing speeds of 55 m.s^−1^, 150 m.s^−1^, and 300 m.s^−1^ corresponding to coating melted over distances equal to 0.6, 0.9, and 1.0 µm, respectively. The standard deviation of the measured melted Cu coating lengths (0.6–1 µm) are indicated with the vertical dotted lines.

Such pattern is often found in metallic glasses^32–34^, polymers^35^, and liquid interfaces^36^. It was also hardly reported in the case of nanocrystalline/amorphous composite microstructure as the Taylor meniscus instability is caused by the penetration into a viscous layer of a less viscous material like air^31^, but it was never reported for crystalline metals. Consequently, the presence of such pattern on the fracture surface of the investigated alloy implies the development of a locally highly viscous state. A local crystalline-amorphous transition could induce the formation of Taylor patterns, however no amorphous transition was observed by high-resolution TEM underneath the fracture surface, as presented in Supplementary Fig. 3. A similar vein pattern was observed on the fracture surface of a TRIP $\beta$ metastable Ti alloy^37^, mistakenly identified as shallow dimples, and on the fracture surface of a nanostructured Ti alloy failing by shear banding^38^, even though it was not for the same conditions of testing or the same level of deformation. It can therefore be anticipated that the original fracture mechanism of Ti-12Mo (wt.%) is not restricted to this alloy but is shared by more $\beta -$ metastable Ti alloys. It can also be argued that the presence of Taylor patterns on the fracture surface finds its origin in a strong localization of the deformation in a shear band and a local thermal softening. A local remelting is also indicated by the presence of droplets on the Taylor patterns, pointed out by white arrows in Fig. 4e. More micrographs of the Taylor meniscus patterns are provided in Supplementary Fig. 4.

### Fusible coating method

The fusible coating method originally proposed by Lewandowski et al.^39^ was used to determine the temperature rise related to the fracture process. The frontier of the melting zone of a deposited coating provides a good indication of the temperature attained locally. A simple one-dimensional differential equation for heat conduction (see Supplementary Equations (1a) and (1b)) was then used to estimate the distribution and time variation of temperature inside the fracture zone. Figure 5a shows that a 50-nm-thick Cu coating deposited on the tensile specimen prior to straining melted near the fracture surface. Two zones can be distinguished: the non-melted region stretched by the deformation of the tensile specimen (consequently divided into several “islands”); and the melted region that can be distinguished owing to its smoother appearance. The coating melted over a total width *l* of 1.1 ± 0.3 µm, i.e. along the tensile axis, as indicated in Fig. 5a. A correction must be applied to obtain the smallest distance $x$ between the melted region and the fracture surface: $x = \cos \left( {\frac{{\uppi }}{4}} \right)l\,=\,0.8 \pm 0.2 {\mathrm{\mu} m}$. The melted region can itself be divided into two zones as observed in Fig. 5a: a first zone $h = 0.7 \pm 0.1 {{\mu}\mathrm{m}}$, where the specimen was deformed the most and which could correspond to the location of the shear band; and a second zone where the Cu coating presents a smaller wettability with the Ti alloy substrate, as the wettability of the melted coating decreases with the decrease of temperature.

## Discussion

The outstanding ductility of generic $\beta$-metastable Ti-12Mo alloy (wt.%) is demonstrated both in terms of resistance to plastic localization and resistance to damage accumulation and cracking. As already developed in earlier works^1,2^, a large resistance to plastic localization was observed due to the high strain hardening caused by the simultaneous TRIP and TWIP effects, resulting in a large true uniform strain. When Considere necking condition is reached at a true strain of 0.3, the alloy can be considered as nanostructured. The creation of a high density of interfaces can indeed accelerate the process of nanostructuration as highlighted by Zafari et al. for TRIP Ti-5553 alloy, where a nanostructure is obtained after a true strain of 3, while an ultra-fined grain microstructure is obtained only after a true strain of 121 for Ti-20Mo (wt.%) alloy deforming by slip^40^. However, the work hardening in Ti-12Mo alloy (wt.%) at a true strain of 0.3 is still large enough to prevent catastrophic failure and a diffuse neck forms. Intensification of the plastic localization at a true strain of about 1.0 brings the formation of a few thin shear bands, in which the final fracture occurs. Even though the large work hardening of Ti-12Mo alloy has postponed this intense localization of the deformation to a large level of strain, it is not enough to ensure a large ductility at fracture as resistance to cracking in metals is controlled by the weakest resistance amongst plastic localization and resistance to damage. In a TRIP–TWIP steel, Wang et al.^19^ proposed that the presence of nanotwins increases the damage resistance of the alloy due to the confinement of the nucleated nanovoids. In the present work, the resistance to damage is even more efficient as the damage nucleation itself is only activated at very large levels of deformation. The interfaces between the martensite, the $\beta$ phase, and the twin boundaries do not constitute weak spots. Otherwise, multiple cavities would have nucleated earlier and more homogeneously. This extraordinary resistance to damage nucleation results in a fracture mechanism dominated by the plastic localization rather than the nucleation, growth and coalescence of cavities. Associated to a large work hardening, it leads to a large true fracture strain ${\it{\epsilon }}_{\mathrm{f}}$, a large work of fracture $W_{\mathrm{f}}$ and a very large estimated fracture toughness $J_{{\mathrm{IC}}}$.

The unusual flatness of the fracture surface can be explained by the development of damage inside a localized deformation band shearing through the entire section of the tensile specimen. The nucleated cavities are all aligned within the bands, and the final thickness of the dimples corresponds to half of the localized band thickness, suggesting that the entire damage mechanism, from nucleation to coalescence, occurs within these bands. Observation of Taylor instability patterns together with the smooth cap of the dimple walls and the presence of droplets on the fracture surface constitute concomitant evidences of a large increase of the temperature up to melting, thus of a viscous liquid state where fracture occurs. Moreover, a local large increase of temperature could trigger dynamic recrystallization and explain the presence of small equiaxed grains and nanoprecipitates of $\omega _{ath}$ inside the shear band (see Fig. 2e–f).

A physically-based model has been developed in order to relate the measured width of the melted coating region with the temperature rise associated to plastic dissipation. As a quasi-static deformation rate was used during the deformation, no significant temperature rise could occur before the localization of the deformation within the shear bands. The heat rate, $\dot \omega$ [$\mathrm{kJ}.{\mathrm{m}}^{ - 3}.\mathrm{s}^{ - 1}$], can therefore be estimated from the mechanical work dissipated during shearing at 45° from the tensile axis through the shear band:

1 $$\dot \omega = \beta \tau _{\mathrm{f}}\dot \gamma ,$$

where $\beta$ is the fraction of mechanical work dissipated into heat (taken here as 0.9); $\tau _{\mathrm{f}} = \frac{{\sigma _{\mathrm{f}}}}{2}$ (=640 MPa) is the true shear stress at fracture computed from the macroscopic fracture stress measurement; and $\dot \gamma = \frac{{v_s}}{{2h}}$ is the true shear strain rate, with $v_s$ being the shearing velocity and *h* is the half-thickness of the shear band (taken as 0.5 µm, the corrected width of the first zone as indicated in Fig. 5a). This heat rate $\dot \omega$ is produced during the shearing time $\delta t$, which is the ratio between the shear offset measured on the fracture surface (27 µm) and the shearing speed $v_{\mathrm{s}}$. The only unknown parameter of the model that describes the temperature rise is therefore the shearing speed $v_{\mathrm{s}}$. This parameter is identified to bring the best correspondence with the width of the melted coating zone. Two boundaries can however be set for the shearing speed. The minimum shearing speed *ν*_s_ is set by the applied straining rate $v_{{\mathrm{applied}}}:v_{\mathrm{s}} > \frac{{v_{{\mathrm{applied}}}}}{{\cos \pi /4}} \sim 2.10^{ - 5}m.s^{ - 1}$. The maximum shearing speed on the other hand can be estimated as 10% of the maximum speed of sound in the material^41^, usually set as the Rayleigh wave speed (i.e. the maximum speed of sound at the surface of the material). The Rayleigh wave speed for pure titanium (2958 m.s^−1^) is used here to estimate the upper bound of the shearing speed: $v_{\mathrm{s}} < 0.1c_{{\mathrm{SR}}}^{{\mathrm{Ti}}}$ ~ 300 m.s^−1^.

Following Wang et al.^42^, the model is analyzed and solved in Supplementary Eqs. (1)–(3). Figure 5b presents the profiles of the highest temperature as a function of the distance $x$ from the fracture surface for different shearing velocities. The melting temperature of the coating $(T_{\mathrm{m}}^{{\mathrm{Cu}}})$ is indicated, together with the melting temperature of the investigated alloy $(T_{\mathrm{m}}^{{\mathrm{Ti}} - {\mathrm{Mo}}})$ and the minimum temperature needed for dynamic recrystallization $(0.4T_m^{Ti - Mo})$. The estimated range of shearing speed needed to melt the Cu coating over a distance of 0.6–1.0 µm is 55–300 m.s^−1^, which corresponds to a maximum temperature reached at the fracture surface in the range of 1250–2450 °C. This temperature range fits with the melting temperature of Ti-12Mo alloy, explaining the melted features and the Taylor meniscus pattern observed on the fracture surface. Moreover, the estimated temperature distribution also correlates quite well with the dynamic recrystallization occurring within the localized shear bands, as a temperature over 0.4 *T*_m_ is predicted to be reached over a distance of 2 µm from the center of the shear band. The important temperature rise observed in the Ti-12Mo alloy is linked to its high shear cracking velocity and to its small thermal diffusivity: 200 cm^2^.s^−1^ for Ti-12Mo at room temperature, compared with 400 cm^2^.s^−1^ for 316 L stainless steel, 1300 cm^2^.s^−1^ for 1018 steel, or 6000 cm^2^.s^−1^ for 7xxx Al alloys. A small thermal diffusivity is also measured in other Ti alloys: 220 cm^2^.s^−1^ for Ti-6-6-2 or 290 cm^2^.s^−1^ for TA6V. However, in these alloys, the smaller damage resistance induces a damage-dominated ductile failure and prevents the unusual fracture sequence observed in the Ti-12Mo alloy. The same fracture mechanism involving local melting and dynamic recrystallization could nevertheless happen in many other highly ductile Ti alloys including the recently designed TRIP–TWIP Ti alloys^3–5,7,43^ as implied by the similar fracture surface of a TRIP $\beta$-metastable Ti alloy^37^, opening a path for fracture properties improvements.

In conclusion, the Ti-12Mo alloy shows an outstanding capacity of dissipating mechanical work during deformation and fracture by combining the damage resistance and failure mechanisms of very ductile materials with the large strain-hardening capacity of TRIP–TWIP alloys providing strength at large strains. The large ductility is due to an extraordinary high resistance to damage nucleation, bringing the full use of the large strain hardening capacity until the localization of the deformation at 45° from the tensile axis into few shear bands. A large temperature rise up to 1250–2450 °C is estimated by the fusible coating method in the center of the shear band during its fracture, causing a local melting of the alloy at the fracture surface and dynamic recrystallization inside the shear band demonstrated by the re-precipitation of ($\omega _{{\mathrm{ath}}}$) in equiaxed *β* grains.

## Methods

### Alloy processing

Ingot of Ti-12 wt.% Mo was processed from commercially pure titanium and molybdenum by the self-consumable melting technique. The chemical composition after casting, measured by Inductively Coupled Plasma (ICP), gives Ti-11.8Mo-0.02Fe-0.01Cu-0.0115 O (in wt.%). A 10 mm thick slice of the ingot was homogenized at 900 °C during 15 min, followed by water quenching. It was then cold rolled down to 1.1 mm, corresponding to a reduction level of 89%. A recrystallization treatment was carried out at 900 °C for 15 min in high-purity Ar atmosphere, followed by water quenching to retain the fully $\beta$ microstructure at room temperature. It is worth noting that nano-sized athermal $\omega _{{\mathrm{ath}}}$ phase is also present in the microstructure, as shown in previous work^1^.

### Material characterization

Uniaxial tension was carried out at 1 mm.min^−1^ on 4 flat tensile specimens with a calibrated gauge length of 27 mm and gage section of 1.1 × 6.1 mm², and, at the same displacement rate, on 2 double edge notched tensile (DENT) specimens of dimensions 0.9 × 20 × 70 mm³, with 7 mm long and 300 µm thick edge notches on each side. Pre-cracks with an opening of <140 µm were produced at the tip of the notches of one DENT specimen with a razor blade and pre-cracks with an opening of less than 1 µm were produced by fatigue on the second DENT specimen. $\delta _{\mathrm{c}}$ was estimated from the difference between the opening of a blunted pre-crack, taken at 45° from the crack, and the opening of a new crack, for a limited new crack length of 400 µm, following Pardoen and Delannay^44^. The opening of the blunted crack and of the new crack were measured five times through the thickness by polishing two cracked DENT specimens. SEM was used to characterize the fracture surfaces and the melted Cu coating regions. A TEM sample was lifted from the localized band using a dual-beam focused ion beam (FIB) instrument, and was characterized using TEM operating at 200 kV. X-ray computed tomography was carried on four cylinders of 300 µm in diameter, and minimum 2 mm long cut from the fracture surface along the tensile direction, with a voxel size of 0.3 µm. The micrographs were analyzed by ImageJ Fiji^45^: the initial images were transformed into binary images by setting a threshold chosen to enhance the contrast between the matrix and the crack coming from the fracture surface, and the ImageJ algorithm “despeckle” was used to remove the noise. The height of the walls of the dimples was measured by white light interferometry, with a depth resolution of 89 nm. The density was measured by Archimedes’ principle in water. The specific heat capacity was measured by differential scanning calorimetry under Ar atmosphere. The thermal diffusivity ${\upalpha}$ was measured by the laser flash technique, in agreement with the ASTM E-1461 standard.

### Cu coating

Tensile specimens were polished down to 1 µm diamond paste and OPS suspension. A 50-nm-thick coating Cu was deposited by Physical Vapor Deposition at a rate of 1 Å/s.

## Supplementary information

Supplementary information

## Data Availability

The datasets generated during and/or analysed during the current study are available from the corresponding author on reasonable request.
