# Supplementary material for: High temperature rise dominated cracking mechanisms in ultra-ductile and tough titanium alloy
Source: Nat Commun. 2020 Apr 30;11:2110. doi: 10.1038/s41467-020-15772-1 (PMC7193587; doi:10.1038/s41467-020-15772-1)
Supplement: Supplementary file 1 — Supplementary information [file 41467_2020_15772_MOESM1_ESM.pdf]

Supplementary Information

High temperature rise dominated cracking mechanisms in ultra-ductile and tough titanium alloy

Choisez et al.

Supplementary Figure 1 – Tomography analysis underneath the fracture surface

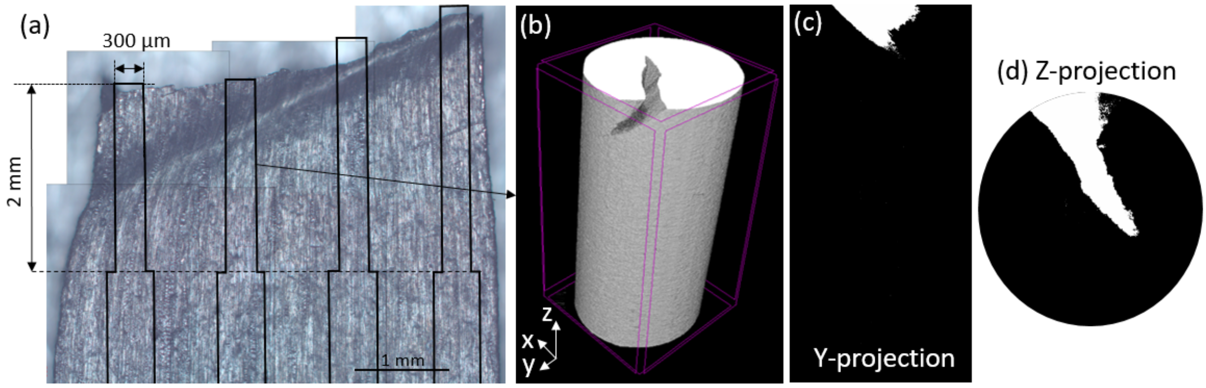

Supplementary Figure 1 X-ray computed tomography. (**a**) Representation of the location of the cylinders machined by electrical discharge machining (EDM) from the fractured specimen (**b**) 3D reconstruction of the second cylinder characterized by tomography. (C) Projection of the images in the y plane and (**d**) in the z plane. The voids are represented in white and the material in black. No voids can be observed.

Figure 1 presents the X-ray computed tomography analysis for one of the 4 cylinders taken underneath the fracture surface, with the cutting outline represented in Figure 1(a). All 4 cylinders show the same result. Figure 1(b) presents the 3D reconstruction, Figure 1(c) the projection of all the images in the Y-plane and Figure 1(d) the projection in the Z-plane. The minimum intensity along the planes was kept for the projection, i.e. all the voids detected through the section are presented in white. The penetration of the crack from the fracture surface inside the tensile specimen can clearly be observed, while no other cavities can be seen.

Supplementary Figure 2 - SEM micrograph of a tensile specimen at high level of strain

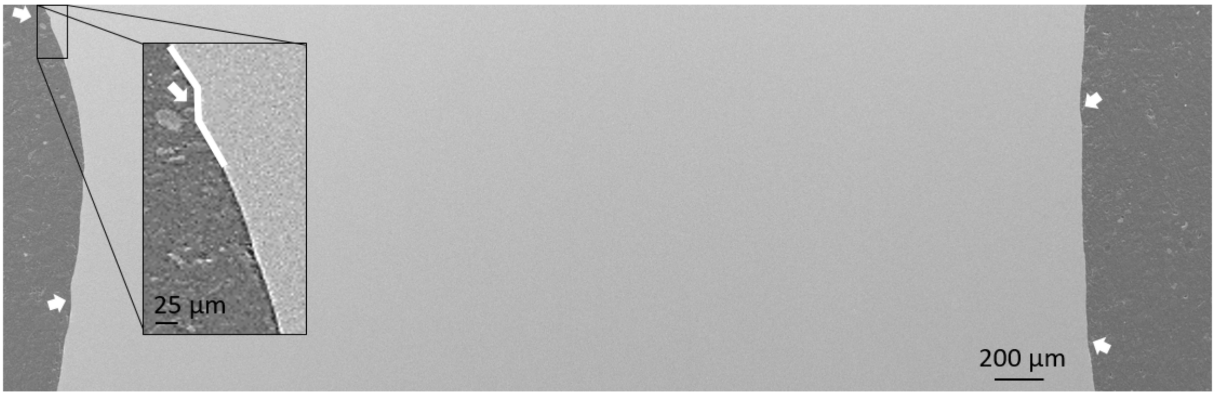

Supplementary Figure 2 SEM micrograph of a tensile specimen deformed to a true strain of 1.03. A tensile specimen was deformed to a true strain close to the fracture strain and polished through its thickness for damage evaluation. The true strain was evaluated from the smallest section of the tensile specimen, measured by white light interferometry before polishing. The formation of small shear steps are indicated by the white arrows, and highlighted in the magnified image.

Figure 2 presents the section of a tensile specimen deformed to a true strain of 1.03, i.e. close to the true fracture strain $\epsilon_f = 0.93 \pm 0.10$. The tensile test was stopped before fracture to evaluate the onset of damage nucleation. While (through-thickness) shearing initiated as highlighted by the shear steps at the edge of the specimen showed by the white arrows in Fig. 2, no cavity can be observed inside the tensile specimen.

Supplementary Figure 3 - TEM analysis of the microstructure below the facture surface

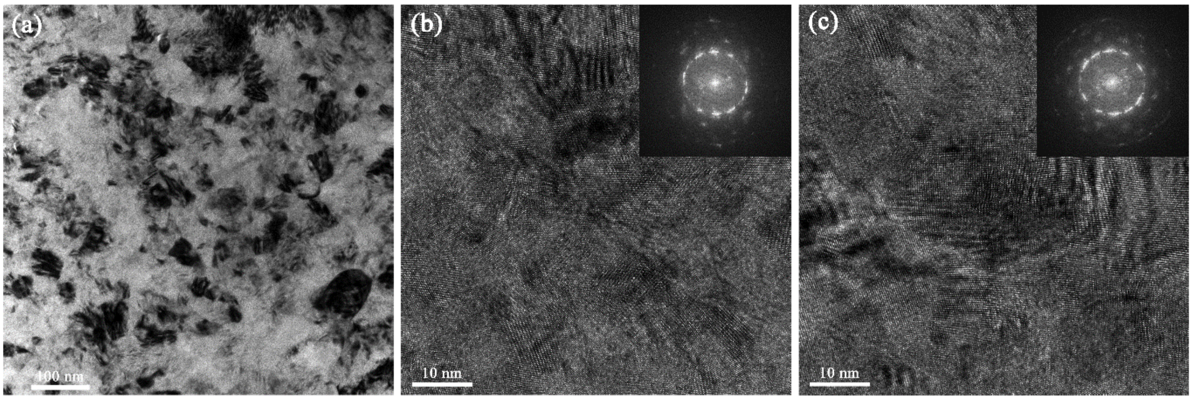

Supplementary Figure 3. High resolution TEM underneath the fracture surface. (a) BF-TEM micrograph of the small equiaxed grains underneath the fracture surface. **(b)** and **(c)** HRTEM micrographs from **(a)**.

The presence of crystalline-amorphous transition was investigated underneath the fracture surface (Figure 3a) by HRTEM. Only crystalline features were observed as evidenced in the HRTEM images of Figures 3b and c as well as in the FFT insets. Clear amorphous zones cannot be observed.

Supplementary Figure 4 - Additional SEM micrograph of the fracture surface

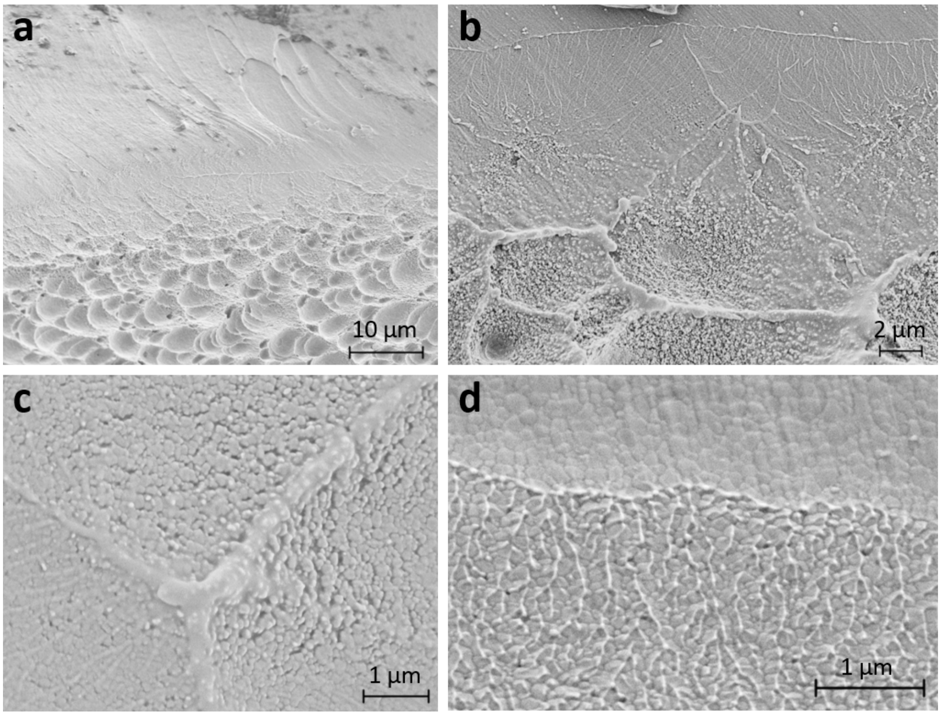

Supplementary Figure 4 SEM micrographs of the Taylor meniscus pattern at multiple length-scales. (a) Overview of the formation of Taylor patterns between a stretched surface and dimples. **(b)** Transition between the Taylor patterns and the granular dimples. **(c)** Magnified view of the junction between three Taylor patterns, which appears melted and resolidified as the wall of the dimples in **(b). (d)** Magnified view of the transition between the stretched surface and the Taylor patterns.

Figure 4(a) presents an overview of the formation of Taylor meniscus patterns between the stretched surface and the dimples, on the edge of the fracture surface. Figure 4(b) and (d) present the transition

between the Taylor patterns and the dimples, and between the Taylor patterns and the stretched surface, respectively. A magnified micrograph of the junction between different Taylor patterns is shown in Fig. 4(c). The junction has the same viscous appearance as the walls of the dimples in Fig. 4(b).

Supplementary Material 5 - Solution of the heat diffusion equation

Following the work of Wang *et al.* [1], the one-dimensional heat diffusion equation used in Lewandowski *et al.* [2] was improved by taking into account the thickness of the shear band and the shearing time during which the heat is produced, allowing a more accurate estimation of the temperature distribution near the fracture surface.

Several hypotheses are made to develop the heat diffusion equation:

- the heat is produced homogeneously in the shear band and during the shearing time;
- a Cartesian infinite regime is chosen, i.e. the temperature of the specimen far from its production site will not be influenced, as the temperature seems to vary only locally;
- the only heat transfer considered is the diffusion of heat through the alloy, which is the dominant mechanism;
- A one-dimensional heat equation is used as the heat will diffuse mainly perpendicularly to the shear band;
- The heat associated to potential phase transformations are not taken into account.

During the shearing time δt, the heat diffusion equation can be divided into two parts (inside the shear band (1a) and outside the shear band (1b)):

$$\frac{\partial \Delta T}{\partial t} = \alpha \frac{\partial^2 \Delta T}{\partial x^2} + \dot{\omega}, \qquad \text{for } x < h, 0 < t < \delta t \qquad (1a)$$

$$\frac{\partial \Delta T}{\partial t} = \alpha \frac{\partial^2 \Delta T}{\partial x^2}, \qquad \text{for } x > h, 0 < t < \delta t \qquad (1b)$$

where $T(x,t)$ is the temperature variation with the distance x from the center of the shear band and the time t; α is the thermal diffusivity [m².s^{-1}]; $\dot{\omega}$ is the heat release rate [J.m^{-}3.s^{-1}] and h is the half thickness of the shear band [m].

The resolution of these equations for a zero initial temperature gives [3]:

$$\Delta T = \frac{\dot{\omega} t}{\rho C_\mathrm{p}}\left[1 - 2i^2erfc\left(\frac{h-x}{2\sqrt{\alpha t}}\right) - 2i^2erfc\left(\frac{h+x}{2\sqrt{\alpha t}}\right)\right] \quad \text{for } x < h, 0 < t < \delta t \qquad (2a)$$

$$\Delta T = \frac{\dot{\omega} t}{\rho C_\mathrm{p}}\left[2i^2erfc\left(\frac{x-h}{2\sqrt{\alpha t}}\right) - 2i^2erfc\left(\frac{h+x}{2\sqrt{\alpha t}}\right)\right] \quad \text{for } x > h, 0 < t < \delta t \qquad (2b)$$

Where $i^2erfc(x) = 0.25\left[(1+2x^2)erfc(x) - \frac{2}{\sqrt{\pi}}x\exp(-x^2)\right]$.

The physical properties of Ti-12wt.% Mo involved in the heat diffusion equations are the density ρ, the specific heat capacity C_p and the thermal diffusivity α, measured as 4869 kg.m^{-3} (at 293K), 700 J.kg^{-1}.K^{-1} (at 1273K) and 691 cm².s^{-1} (at 1273K), respectively (see Methods in the main manuscript). The values of specific heat capacity and thermal diffusivity at 1273K were used as a compromise between the heat diffusion at high temperature (up to the melting temperature at about 2000K) and at room temperature, to take into account the better heat diffusion capacity of the material when the temperature increases.

For $t > \delta t$, Equation (1b) can be solved with an initial distribution of temperature $f(x)$ corresponding to the distribution of temperature at the end of the shearing stage [4]:

$$\Delta T = \frac{1}{\sqrt{4\pi\alpha t\prime}} \int f(u) \exp\left(-\frac{(x-u)^2}{4\alpha t\prime}\right) du \qquad \text{for } t' > 0, t = t' + \delta t \qquad (3)$$

References

1. Wang, J. G. *et al.* How hot is a shear band in a metallic glass? *Mater. Sci. Eng. A* **651**, 321–331 (2016).

2. Lewandowski, J. J. & Greer, A. L. Temperature rise at shear bands in metallic glasses. *Nat. Mater.* **5**, 15–18 (2006).
